# Supplementary material for: Smooth Interpolating Curves with Local Control and Monotone Alternating Curvature
Source: Comput Graph Forum. 2022 Oct 6;41(5):25–38. doi: 10.1111/cgf.14600 (PMC9827861; doi:10.1111/cgf.14600)
Supplement: Supplementary file 1 — Supplement Material [file CGF-41-25-s001.zip › Local-Smooth-Interpolating-MonoCurvature/extern/clothoids/submodules/Utils/docs/api-cpp/program_listing_file_TicToc.cc.html]

Program Listing for File TicToc.cc — Utils v1.2

### Navigation

- index
- toc
- Utils »
- Program Listing for File TicToc.cc

# Program Listing for File TicToc.cc¶

↰ Return to documentation for file (`TicToc.cc`)

```
/*--------------------------------------------------------------------------*\
 |                                                                          |
 |  Copyright (C) 2017                                                      |
 |                                                                          |
 |         , __                 , __                                        |
 |        /|/  \               /|/  \                                       |
 |         | __/ _   ,_         | __/ _   ,_                                |
 |         |   \|/  /  |  |   | |   \|/  /  |  |   |                        |
 |         |(__/|__/   |_/ \_/|/|(__/|__/   |_/ \_/|/                       |
 |                           /|                   /|                        |
 |                           \|                   \|                        |
 |                                                                          |
 |      Enrico Bertolazzi                                                   |
 |      Dipartimento di Ingegneria Industriale                              |
 |      Universita` degli Studi di Trento                                   |
 |      Via Sommarive 9, I-38123 Povo, Trento, Italy                        |
 |      email: enrico.bertolazzi@unitn.it                                   |
 |                                                                          |
\*--------------------------------------------------------------------------*/


#include "Utils.hh"

#ifdef UTILS_OS_WINDOWS

#include <windows.h>

namespace Utils {

  #define TOINT64(A) (static_cast<int64_t>(A.HighPart) << 32) | A.LowPart

  TicToc::TicToc() : m_elapsed_time(0) {
    LARGE_INTEGER frequency;
    QueryPerformanceFrequency(&frequency);
    m_frequency = frequency.QuadPart;
    tic();
  }

  void
  TicToc::tic() {
    LARGE_INTEGER t1;
    QueryPerformanceCounter(&t1);
    m_t1 = t1.QuadPart;
  }

  void
  TicToc::toc() {
    LARGE_INTEGER t2;
    QueryPerformanceCounter(&t2);
    m_t2 = t2.QuadPart;
    m_elapsed_time = (m_t2 - m_t1) * 1000.0 / m_frequency;
    ;
  }

  void
  sleep_for_seconds( unsigned s ) {
    Sleep(DWORD(s) * 1000);
  }

  void
  sleep_for_milliseconds( unsigned ms ) {
    Sleep(DWORD(ms));
  }
}

#else

namespace Utils {

  void
  TicToc::tic()
  { m_start_time = clock::now(); }

  void
  TicToc::toc() {
    m_stop_time    = clock::now();
    m_elapsed_time = std::chrono::duration_cast<elapsed_resolution>(m_stop_time - m_start_time);
  }

  typename TicToc::real_type
  TicToc::elapsed_s() const
  { return 1e-6*m_elapsed_time.count(); }

  typename TicToc::real_type
  TicToc::elapsed_ms() const
  { return 1e-3*m_elapsed_time.count(); }
}

#endif
```

### Quick search

### Table of Contents

- rang - Colors for your Terminal
- Zstr
- C++ API

«
hide menu

menu
sidebar
»

### Navigation

- index
- toc
- Utils »
- Program Listing for File TicToc.cc

© Copyright 2021, Enrico Bertolazzi.
Created using Sphinx 4.2.0.
